# A controlled trial of the Litebook light-emitting diode (LED) light therapy device for treatment of Seasonal Affective Disorder (SAD)

**DOI:** 10.1186/1471-244X-7-38

**Published:** 2007-08-07

**Authors:** Paul H Desan, Andrea J Weinstein, Erin E Michalak, Edwin M Tam, Ybe Meesters, Martine J Ruiter, Edward Horn, John Telner, Hani Iskandar, Diane B Boivin, Raymond W Lam

**Affiliations:** 1 Department of Psychiatry, Yale University, PO Box 208068, New Haven, CT 06520-8068, USA; 2 Mood Disorders Centre, Department of Psychiatry, University of British Columbia, Vancouver, British Columbia, Canada; 3 University Medical Center Groningen, Groningen, The Netherlands; 4 Royal Ottawa Mental HealthCentre, Ottawa, Ontario, Canada; 5 Centre for Study and Treatment of Circadian Rhythms, Douglas Hospital Research Centre, Montreal, P. Quebec, Canada

## Abstract

**Background:**

Recent research has emphasized that the human circadian rhythm system is differentially sensitive to short wavelength light. Light treatment devices using efficient light-emitting diodes (LEDs) whose output is relatively concentrated in short wavelengths may enable a more convenient effective therapy for Seasonal Affective Disorder (SAD).

**Methods:**

The efficacy of a LED light therapy device in the treatment of SAD was tested in a randomized, double-blind, placebo-controlled, multi-center trial. Participants aged 18 to 65 with SAD (DSM-IV major depression with seasonal pattern) were seen at Baseline and Randomization visits separated by 1 week, and after 1, 2, 3 and 4 weeks of treatment. Hamilton Depression Rating Scale scores (SIGH-SAD) were obtained at each visit. Participants with SIGH-SAD of 20 or greater at Baseline and Randomization visits were randomized to active or control treatment: exposure to the Litebook LED treatment device (The Litebook Company Ltd., Alberta, Canada) which delivers 1,350 lux white light (with spectral emission peaks at 464 nm and 564 nm) at a distance of 20 inches or to an inactivated negative ion generator at a distance of 20 inches, for 30 minutes a day upon awakening and prior to 8 A.M.

**Results:**

Of the 26 participants randomized, 23 completed the trial. Mean group SIGH-SAD scores did not differ significantly at randomization. At trial end, the proportions of participants in remission (SIGH-SAD less than 9) were significantly greater (Fisher's exact test), and SIGH-SAD scores, as percent individual score at randomization, were significantly lower (t-test), with active treatment than with control, both in an intent-to-treat analysis and an observed cases analysis. A longitudinal repeated measures ANOVA analysis of SIGH-SAD scores also indicated a significant interaction of time and treatment, showing superiority of the Litebook over the placebo condition.

**Conclusion:**

The results of this pilot study support the hypothesis that light therapy with the Litebook is an effective treatment for SAD.

**Trial registration:**

Clinicaltrials.gov: NCT00139997

## Background

Seasonal Affective Disorder (SAD, winter depression) is a well-recognized form of recurrent depressive disorder, characterized by typical and atypical (increased appetite, weight, sleep and fatigue) depressive symptomatology and a distinct seasonal nature [1,2]. SAD is thought to be related to natural seasonal variations in light levels. Bright light therapy – exposure of the patient each morning to bouts of artificially produced high intensity light – has been shown to produce amelioration of depressive symptoms. Over 70 trials addressing the efficacy of light therapy have now been conducted, including 2 large controlled trials [3,4] which demonstrated clear efficacy. Light therapy was found to be similar in efficacy to treatment with fluoxetine in a large controlled trial [5]. Several meta-analyses have found that light treatment is effective for SAD [6-8]. While light therapy appears to be an efficacious form of treatment, the traditional mode of delivery via a relatively large and bulky light box can be cumbersome for patients. Finding easier and briefer forms of treatment has been a major goal of the field.

Light therapy using light-emitting diodes (LEDs) may offer advantages over conventional light boxes based on fluorescent or incandescent sources. First, recent data indicate that the human circadian rhythm system is most sensitive to light with wavelength in the range 450 – 480 nm [9-11]. LEDs can be selected to emit light with energy concentrated in this range, while fluorescent and incandescent sources emit across the visible spectrum. Although the role of the circadian rhythm system in the pathophysiology of SAD is unclear [12], one study has shown that LED-generated blue light (398 lux, peak energy output around 468 nm) was more effective than LED-generated red light (23 lux, peak output around 654 nm) [13]. Secondly, LEDs are more efficient and lighter than traditionally used fluorescent tubes, and may permit significantly smaller and lighter treatment devices. The aim of the present study was to conduct a randomized placebo-controlled trial to test the efficacy of a white LED device whose light emission was relatively concentrated in shorter wavelengths (the "Litebook", The Litebook Company Ltd., Alberta, Canada). Since negative ion generators have been reported to be effective in treatment of SAD [14], a "credible placebo" design similar to that of Eastman and colleagues [4], in which an inactivated negative ion generator was used as a "no light" control condition, was employed. The results suggest that treatment with the Litebook LED device is an effective treatment for SAD.

## Methods

### Study Protocol

This is a multi-center, randomized, double-blind, parallel-group clinical trial of light therapy for participants with SAD (winter type). Participants were seen at a Baseline Visit, a Randomization Visit, and after 1, 2, 3 and 4 weeks of treatment. Participants who appeared to meet the inclusion criteria and not meet exclusion criteria at the Baseline Visit were invited to return in 1 week for a Randomization Visit. At this visit participants who continued to meet study criteria were issued either an active light treatment device or a placebo inactivated ion generator. Participants were seen at weekly intervals during 4 weeks of treatment. Participants were enrolled between October 1 and March 1 to reduce confounding effects of natural remission as expected in the spring.

Severity of depressive symptoms was rated at each visit using a 24-item SIGH-SAD, a scripted version of the Hamilton Depression Rating Scale [15] modified to reflect better the atypical symptomatology of SAD [16]. This version of the SIGH-SAD consists of the HDRS 17-item scale plus the first 7 atypical items (i.e., excluding Reverse Diurnal Variation). At the Randomization Visit and the subsequent 4 visits, SIGH-SAD ratings were carried out by a clinician blinded to the assigned treatment device. The blinded clinician also completed a systematic inquiry about any adverse events. A separate unblinded clinician dispensed and demonstrated the treatment device at the Randomization Visit, and was available at subsequent visits if required.

The study was conducted at 5 sites, in New Haven (USA), Vancouver, Montreal and Ottawa (Canada), and Groningen (The Netherlands). The research protocol was approved by applicable institutional review boards and met standards established by the Helsinki Declaration, and participants signed appropriate consent forms. The trial was registered at the U.S. National Institutes of Health clinical trials database [17].

### Participants

Participants were recruited through media advertisements or professional referrals, screened by experienced interviewers by telephone, and if appropriate invited for a Baseline Visit. At this visit, participants received a full psychiatric evaluation, physical exam, urine toxicology for commonly abused substances, and urinary beta-HCG for female participants. Participants were required to be between ages 18 and 65, to have a DSM-IV diagnosis of SAD (major depressive episode, with seasonal pattern, winter type [18] and to have a SIGH-SAD score of 20 or greater. Diagnosis was established with the Structured Clinical Interview for DSM-IV (SCID) [19]. Participants also completed the Morningness-Eveningness Questionnaire (MEQ), a measure of preference for activity in the early or late part of the day [20].

Participants were told that the study involved treatment with either a new light treatment device or a negative ion generator, that both types of treatment were experimental, and that the study was placebo-controlled. In particular, participants were told that one half of the devices in the study were modified in such a way that the investigators did not expect the device to be efficacious. In order to demonstrate informed consent, participants had to demonstrate understanding that if they participate they have a one in two chance of being assigned to treatment expected to be inactive for 4 weeks.

Exclusion criteria were: significant medical illness, any retinal disease or medical disorder associated with retinal disease; pregnancy; use of photosensitizing medications, mood-altering medications, light therapy or other treatment for SAD within 1 week of the Baseline Visit (except within 4 weeks in the case of pharmacological antidepressant agents); initiation of psychotherapy within 3 months of the Baseline Visit, except where terminated by the participant prior to this visit; current organic mental disorder, panic disorder, anorexia or bulimia nervosa, obsessive-compulsive disorder or posttraumatic stress disorder; a history of any psychotic disorder or bipolar I disorder (history of manic episode); a history of substance use disorder not in full remission for at least one year; unstable sleep or mood patterns (such as severe premenstrual syndrome); previous unsuccessful trial of light therapy with an accepted device for at least 2 weeks; inability to provide informed consent; poor likelihood of complying reliably with study requirements; suicidal risk or other factor making trial participation clinically inappropriate. Participants were required to have a habitual sleep onset time before 1 A.M., and a habitual sleep end time before 9 A.M., prior to entry in the trial. Participants were required to agree to avoid other treatments for SAD or excluded medications, alteration of daily schedule to change light exposure, or travel to sunny destinations, to maintain a stable sleep schedule, and if female and potentially fertile to use an appropriate form of contraception during the trial.

### Treatment Devices

At the Randomization Visit, eligible participants were issued an active or control treatment device by the unblinded clinician. Assignment to active or control group was determined by telephone call by the unblinded clinician to the trial sponsor, and was balanced in blocks of 4 for each site and gender. The proper use of the device was demonstrated to the participant by the unblinded clinician. After experiencing the assigned device in operation, the participant completed a brief questionnaire about expectations [21]. Participants were given a tape 20 inches in length to indicate the correct distance from the device.

The active treatment consisted of a Litebook treatment device with 60 LEDs (The Litebook Company Ltd., Alberta, Canada). The 60 LEDs employed in this Litebook model contain emitters which have a spectral emission peak at approximately 464 nm and fluorescent phosphors which provide a broader, secondary spectral peak near 564 nm: of the energy emitted over the range 400 to 700 nm, about 48% is emitted over the range 420 to 508, and 37% is emitted over the range 512 to 616 nm. Collectively the emitted light appears white. This device produces approximately 1,350 lux light at 20 inches. Participants assigned to this device were carefully instructed on aligning the device to illuminate maximally the eyes. An evaluation by an independent consultant physicist confirmed that the Litebook device meets the relevant sets of standards for light exposure safety [22-24].

Control treatment consisted of a negative ion generator, modified to emit no negative ions (SphereOne, Inc., Silver Plume, CO) and to generate a faint high-pitched whine, used at the same distance. Participants using the ion generator were instructed to wear a wrist strap connected to the device to maximize the transfer of negative ions, as this intervention has been found to increase expectations regarding efficacy for the device [3].

Participants were instructed to use the device for 30 minutes each morning, as soon as possible upon arising, and to complete treatment before 8 A.M. Participants were asked to maintain as stable a schedule of sleep and treatment as possible during the trial, and were asked to complete a log of the times of the beginning and end of sleep and of treatment. Participants were asked not to disclose to the blinded clinician which treatment device they were assigned. The blinded study clinician was permitted to reduce the duration of treatment to 15 minutes per day until the next study visit in the event of jitteriness or over stimulation, but this reduction was not required for any participant during the trial.

### Statistical analysis

SIGH-SAD scores were analyzed in both a last observation carried forward (LOCF) analysis, including all 26 participants who were randomized, and an observed cases (OC) analysis, including all 23 participants who completed the trial. Remission was defined as a SIGH-SAD score less than 9. The *a priori *endpoint hypothesis was whether the proportion of participants in remission differed between the active and control treatment groups in the LOCF analysis using the Fisher's exact test. In a secondary analysis, end trial SIGH-SAD scores, as %SIGH-SAD scores consisting of final score as percentage of individual score at randomization, were compared between active and control groups by t test. *Post hoc *comparisons of the proportion of participants in remission and mean %SIGH-SAD score were made at Weeks 1, 2 and 3. Secondary analysis also included a repeated measures ANOVA mixed model with SIGH-SAD as dependent variable and time, treatment, and interaction of time and treatment as fixed effects, including all randomized participants. A variety of models were considered, including participant intercept and slope as random effects, autoregressive time-dependent, compound symmetry or unstructured correlation structures, and possible transformation of time by the log of one plus the week of treatment. The best model was selected by Schwartz Bayesian criterion, but all models indicated a significant interaction of time by treatment. The final model included linear time trend, no random effects, transformed time, and autoregressive correlation structure (SAS PROC MIX procedure, Kenward-Rogers method for degrees of freedom). Statistical assumptions were verified by examination of residuals.

Comparisons between the groups at baseline were made with t-tests in the case of continuous variables and Fisher exact tests in the case of dichotomous variables. Changes in time of sleep or treatment were analyzed with paired t-tests for participants who completed the trial, excluding one participant with incomplete sleep log data. End trial %SIGH-SAD scores were used to assess any relationship between therapeutic response and times of sleep or treatment or other covariate. Statistical analysis was performed with STATVIEW version 5.0.1 and SAS version 9.1.3 (both from SAS Institute, Cary, NC). All results are reported as means ± standard deviations.

## Results

Twenty six participants were randomized into the study, 15 in the active treatment group and 11 in the control treatment group. In the active treatment group, 1 participant withdrew after the visit Week 1 for unclear reasons, possibly related to adverse effects of jitteriness and headache or to travel plans. In the control treatment group, 1 participant was withdrawn after Week 1 due to lack of improvement, and 1 participant was withdrawn after Week 1 due to missed treatments related to a motor vehicle accident. Thus, 23 participants completed the Week 4 visit, 14 in the active treatment group and 9 in the control treatment group. There were no instances of accidental unblinding of the depression rating clinicians during the trial.

Mean SIGH-SAD scores for the active and control treatment groups did not differ significantly at randomization (28.0 ± 5.35 versus 25.1 ± 3.22, respectively; as shown in Table 1). There were no significant differences between the active and control groups in age (44.7 ± 12.3 years versus 47.6 ± 10.8 years), fraction of female participants (64.3% versus 88.9%), fraction of Caucasian participants (85.7% versus 100%; in the active treatment group, 1 participant was Black and 1 participant Hispanic), number of previous episodes of SAD (11.1 ± 9.9 versus 10.6 ± 9.0), age of first SAD episode (30.3 ± 11.6 versus 35.4 ± 13.4), weight (78.4 ± 18.0 kg versus 71.1 ± 14.1 kg), BMI (28.9 ± 6.5 versus 26.1 ± 5.1), expectation scores (3.88 ± 0.70 versus 3.37 ± 0.86), or MEQ scores (51.5 ± 10.2 versus 55.2 ± 6.3).

**Table 1:** SIGH-SAD Outcome Measures at Randomization and after 1, 2, 3 and 4 weeks of treatment.

	Mean SIGH SAD score*		Mean SIGH SAD score As % of randomization score			% Participants in remission (SIGH SAD <9)		
	Active	Control	Active	Control	p value	Active	Control	p value

Randomization	28.0 ± 5.3	25.1 ± 3.2	-	-	-	-	-	-
Week 1	18.6 ± 7.9	19.0 ± 7.2	67.1 ± 26.7	74.0 ± 22.5	0.527	7.1	11.1	0.999
Week 2	14.9 ± 10.0	17.9 ± 4.9	54.5 ± 36.4	71.7 ± 19.5	0.208	28.6	0.0	0.127
Week 3	11.1 ± 10.1	14.9 ± 4.2	39.0 ± 30.7	54.4 ± 17.9	0.080	42.9	11.1	0.176
Week 4	8.7 ± 8.4	13.4 ± 5.4	29.9 ± 25.4	54.4 ± 21.8	0.027**	57.1	11.1	0.040***

Notes: Observed cases analysis: for active treatment n = 14 and for placebo treatment n = 9. * interaction of time and treatment significant in repeated measures ANOVA as noted in text. ** comparison significant at p ≤ 0.05 by t test. *** comparison significant at p ≤ 0.05 by Fisher's exact test.

**Figure 1:**
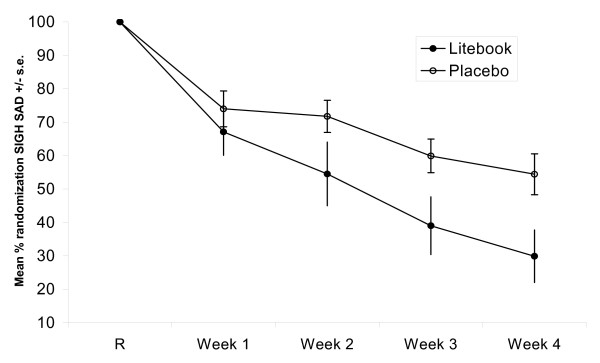
**Mean %SIGH-SAD Score after 1, 2, 3 and 4 weeks of treatment**. The mean SIGH-SAD score, as percent of individual participant value at the Randomization Visit, is shown for participants receiving active (n = 14) and placebo (n = 9) treatment in the observed cases analysis, at the Randomization Visit ("R") and after 1, 2, 3 and 4 weeks of treatment. Error bars indicate standard error of the mean.

SIGH-SAD scores improved in both groups over the 4 weeks of treatment, with active treatment participants showing greater improvement (Table 1, Figure 1). The proportion of participants achieving remission was significantly greater in the intent-to-treat LOCF analysis: 53.3% versus 9.1%, p = 0.036 (the *a priori *endpoint hypothesis of the trial). The proportion of participants achieving remission was also significantly greater with active than control treatment in the OC analysis of all randomized participants: 57.1% versus 11.1%, p = 0.040 (Fisher's exact test; remission defined as SIGH-SAD score <9). There were no significant differences in proportion of participants in remission in pairwise *post hoc *comparisons prior to Week 4.

Mean %SIGH-SAD scores (final SIGH-SAD score as percent of the individual participant score at randomization) were significantly different between the active and control groups at trial end, in both the intent-to-treat LOCF analysis, 34.5% ± 30.47% versus 60.4% ± 23.61%, p = 0.028, and the OC analysis, 29.9% ± 25.4% versus 54.4% ± 21.8%, p = 0.027. Mean %SIGH-SAD scores did not differ significantly in pairwise *post hoc *comparisons prior to Week 4. Analysis of SIGH-SAD scores with a repeated measures ANOVA indicated a significant effect of time (F (1,115) = 71.2, p < 0.0001) and a significant interaction of treatment and time (F(1,115) = 5.30, p = 0.023). These results indicate that the active light treatment condition was significantly superior to the placebo control condition.

There was no significant correlation between expectation scores and therapeutic response measured as final %SIGH-SAD scores: for all participants, r^2 ^= 0.00 (p = 0.86), for participants on active treatment, r^2 ^= 0.07 (p = 0.35), and for participants on control treatment, r^2 ^= 0.03 (p = 0.63). There was no significant correlation between pre-treatment MEQ scores and therapeutic response measured as %SIGH-SAD scores for all participants, r^2 ^= 0.03 (p = 0.45), for participants on active treatment, r^2 ^= 0.00 (p = 0.79), or for participants on control treatment, r^2 ^= 0.00 (p = 0.84).

Times of self-reported sleep start, sleep midpoint, sleep end, and treatment start are shown in Table 2 for all participants who completed the trial, for the week before treatment, the first week of treatment and the last week of treatment in Table 2. There was no significant difference between active and control groups in any of these variables in any week. There were no significant changes in time of sleep start between the baseline week and first week of treatment, or between the first and last week of treatment in either group. The time of sleep end was earlier in the first week of treatment than in the baseline week in both active and control groups (within-group difference significant at p < 0.0001 and p = 0.047, respectively, paired t test), presumably reflecting the need to complete treatment by 8 A.M. as required by the protocol. Between the first and last week of treatment the time of sleep end shifted somewhat later in the active group (p = 0.011, paired t test). Times of sleep midpoint and of treatment start showed a similar pattern to time of sleep end, as might be expected.

**Table 2:** Mean times of sleep and treatment during baseline week and during first and last weeks of treatment.

	Sleep onset time		Sleep midpoint time		Sleep end time	
	Active	Placebo	Active	Placebo	Active	Placebo

Baseline week	23:04 ± 1:01	23:08 ± 0:50	3:06 ± 0:44^1^	3:17 ± 0:59	7:07 ± 0:51^2^	7:26 ± 1:22
First week treatment	23:11 ± 0:45	22:58 ± 0:30	2:48 ± 0:32^1^	2:57 ± 0:46	6:25 ± 0:40^2,3^	6:56 ± 1:14
Last week treatment	23:05 ± 0:49	22:58 ± 0:60	2:52 ± 0:38	2:55 ± 0:55	6:39 ± 0:44^3^	6:52 ± 0:60

	Time of treatment		Time to treatment after midpoint of sleep			

	Active	Placebo	Active	Placebo		

First week treatment	6:42 ± 0:44^4^	7:03 ± 0:45	3:54 ± 0:29^5^	4:03 ± 0:34		
Last week treatment	6:55 ± 0:45^4^	7:07 ± 0:50	4:01 ± 0:28^5^	4:02 ± 0:35		

Notes: Data is included for all participants who completed the trial, n = 14 for active and n = 9 for placebo treatment. No comparison between active and placebo group was statistically significant for any of the variables shown. Comparisons between the first week treatment and the baseline week or last week of treatment that were significant at p ≤ 0.05 in paired t test are indicated by shared superscripts.

There was no significant statistical correlation or apparent relationship between end trial %SIGH-SAD and time of treatment (r = 0.01, p = 0.74), in participants on active treatment who completed the trial. Most participants (11 of 15) received treatment between 6:10 A.M. and 7:40 A.M., and all of these showed a response with final score less than 50% of pre-treatment. There was no significant statistical correlation or apparent relationship between end trial %SIGH-SAD and the interval between time of treatment and sleep midpoint (r = 0.03, p = 0.53), in completing participants on active treatment. Most participants (11 of 15) received treatment beginning between 3:20 and 4:30 hours after sleep midpoint and all but 1 had a final %SIGH-SAD score less than 50% of pre-treatment.

Few treatment-related adverse effects were reported during the trial. In the active treatment group, jitteriness and headache were reported by 1 participant at Week 1, dry mouth and difficulty falling asleep by another participant at Week 1. In the control treatment group, jitteriness was reported by 1 participant at Week 1.

## Discussion

The results of this pilot study suggest that 30 minutes of daily light exposure to the Litebook LED device is efficacious in the treatment of SAD: the *a priori *hypothesis of a difference in remission rate between active and control treatment was supported. The rate of remission in the active group, 57%, was comparable to the remission rate observed by Eastman et al [4] with 1 hour daily use of a 5,000 lux light box (61%). Recent studies have indicated that the human circadian rhythm system is most sensitive to short wavelength light. For example, melatonin secretion is most powerfully inhibited by light with wavelength in the range 450 – 480 nm [9,11], and melatonin rhythms are best shifted by such wavelengths [10]. One study found light with wavelengths around 468 nm more effective in the treatment of SAD than light with wavelengths around 654 nm [13]. The spectral energy distribution of light emitted by the Litebook LED device peaks at about 464 nm, and 48% of its energy is in the range of 420 nm to 508 nm. It is reasonable to hypothesize that the LED device is therapeutically similar to the brighter light box due to this concentration in the short wavelengths. Demonstration that the therapeutic effect of the Litebook device is similar to that of a standard light box would require direct comparison trials.

Therapeutic response with the Litebook device appeared to be gradual, with separation of the active and control groups increasing between 1 and 4 weeks. Our results are similar to those of Eastman et al [4], who observed a significant difference in response rate at Week 3 and 4. Most studies of light therapy for SAD have been 1 or 2 weeks in duration, but gradual onset of response was observed in the 4 week trial by Bauer et al [25], and in the 8 week trial by Lam et al [5]. It is possible that an 8 week trial would have shown a further increased therapeutic response. There is some evidence that trial length may affect speed of therapeutic effect in light therapy, with participants randomized to shorter treatment having a faster response than those randomized to longer treatment [26].

Selection of an appropriate control has been problematic in light treatment research, since, as in the case of some other medical devices, the modality of treatment cannot be "blind". Most such research has used treatment with dim red light as a placebo intervention. There are 2 problems with this approach. First, as the use of bright light is increasingly recognized by the public as a treatment for SAD, participants may become more likely to perceive dim red light as the placebo condition, while participants exposed to bright light may be more likely to conclude they are receiving the active condition. Second, even dim light can affect the circadian rhythm system and may have some positive therapeutic effect [27]. The present study used a "credible placebo" design. A no-light device with a plausible therapeutic mechanism served to control for non-specific behavioral effects of light therapy (e.g., sitting for 30 minutes, waking before 8:00 A.M.). Expectations for the light device were not significantly higher than for the ion generator, and there was no significant correlation between expectation score and therapeutic response.

The relationships between therapeutic response and times of sleep and of treatment are important for theoretical and practical issues. It has been proposed that SAD is related to a phase delay of circadian rhythms, and that light treatment in SAD is effective by advancing circadian rhythms [28]. Terman and colleagues [29] observed a shift to earlier time of sleep midpoint during light treatment for SAD, suggesting a phase advance of circadian rhythm. In the present trial we observed only a shift towards later time of sleep end and midpoint during the 4 weeks of treatment. In the present trial, participants in both active and control groups appeared to move their times of awakening earlier between the pre-treatment week and the first week of treatment, likely because they were required to complete treatment by 8 A.M. Light treatment then appeared to be associated with a small delay in time of awakening during the treatment period. The protocol of the trial may have obscured the ability to observe shifts in circadian phase.

The observations of Terman et al [29] would suggest that response to treatment ought to be strongest about 1.5 – 2 hours after sleep midpoint in a participant with typical 11 P.M to 7 A.M. sleep cycle. The present results do suggest that treatment between 6:10 and 7:40 A.M. in clock time, or between 3:20 and 4:30 hours after sleep midpoint, was effective in alleviating SAD. With the limited number of participants in this trial it is not possible to draw detailed conclusions about the dependence of therapeutic response on time of treatment. Studies with other methodologies, such as that of Murray et al [30], have not observed relationships like those observed by Terman et al [29]. A more detailed version of the phase shift hypothesis suggests that the relationship between times of sleep and time of temperature minimum is critical in the pathogenesis and treatment of SAD [31]. We were unable to measure any such shift in phase angle difference as there was no measure of physiological rhythms in our participants (nor did we measure end trial MEQ, which might serve as a surrogate measure). There was no discernable relationship between MEQ and therapeutic response to light treatment. A study of this size might not be adequate to detect such an association.

Reports of adverse events were rare in the trial and the light treatment was well tolerated by participants. Jitteriness was observed in 1 participant each in the active and control treatment groups. This trial is too small to permit accurate assessment of a difference in the occurrence of this symptom between active and control treatment. No ocular adverse events were observed. Studies with ophthalmological examination before and after treatment have disclosed no harmful effect of treatment with conventional light boxes [32], but such studies have not been conducted with the Litebook device.

In summary, this pilot randomized controlled trial supported the hypothesis that the Litebook LED device is significantly superior to a credible placebo control condition for the treatment of SAD. However, the results of a small-sample clinical trial must be interpreted with caution. A trial with a larger sample size would provide more definitive information about the efficacy and safety of this LED device. A more convenient form of light therapy might lead to increased use of light for SAD and other biological rhythm disturbances.

## Conclusion

At the end of this 4 week randomized, double-blind, placebo-controlled trial, the proportions of participants in remission (SIGH-SAD < 9) were significantly greater, and SIGH-SAD scores (as percent of individual score at randomization) were significantly lower with treatment with the Litebook LED light therapy device than with placebo treatment. A longitudinal repeated measures ANOVA analysis of SIGH-SAD scores also indicated a significant interaction of time and treatment. These results are consistent with the hypothesis that the Litebook device is an effective therapy for SAD. There was no significant correlation between therapeutic response and expectation scores, MEQ scores, or time of treatment expressed as clock time, or as time since the midpoint of sleep. Treatment was well-tolerated, with only transient minor adverse effects.

## Competing interests

PHD and RWL have received research funding and served as consultants for manufacturers of pharmaceutical antidepressants which could be considered alternative therapies for SAD. RWL has received honoraria as a member of the professional advisory board of The Litebook Company Ltd., and holds stock options in the company: he did not participate in the clinical conduct of the trial or the analysis of unblinded data. DBB reports the gift of light treatment devices for other research studies from The Litebook Company Ltd, but has no other potential conflicts of interest. AJE, EEM, EMT, YM, MJR, EH, JE and HI reported no potential conflicts of interest.

## Authors' contributions

The original version of the experimental protocol was written by PHD and EEM, with important input from RWL in the initial design and conduct of the study. PHD served as the overall project principal investigator, and AJW served as the overall research coordinator. EMT, EH, DBB, PHD and YM served as principal investigators at the individual sites. MJR, HI, and JT participated in the clinical conduct of the trial. The final manuscript was written by PHD, with comments from all co-authors, all of whom read and approved the final manuscript.

## Pre-publication history

The pre-publication history for this paper can be accessed here:


